# T-Cell Repertoire Characteristics of Asymptomatic and Re-Detectable Positive COVID-19 Patients

**DOI:** 10.3389/fimmu.2021.769442

**Published:** 2022-01-27

**Authors:** Yizhe Li, Jian Hu, Yongsi Wang, Dongdong Liu, Yaling Shi, Jiaqi Zhang, Yuntao Liu, Dongzi Lin, Jing Lin, Wei Hu, Haolan He, Wei Wang, Wentao Fan, Linlin Li, Dawei Wang, Kejian Wang, Jianhua Xu

**Affiliations:** ^1^Department of Laboratory Medicine, Shunde Hospital of Guangzhou University of Chinese Medicine, Foshan, China; ^2^Department of Laboratory Medicine, The Second Affiliated Hospital of Guangzhou University of Chinese Medicine, Guangzhou, China; ^3^Department of Translational Medicine Research Institute, Guangzhou Huayin Medical Laboratory Center Ltd., Guangzhou, China; ^4^Department of Laboratory Medicine, Guangzhou Eighth People’s Hospital, Guangzhou Medical University, Guangzhou, China; ^5^Emergency Department, The Second Affiliated Hospital of Guangzhou University of Chinese Medicine, Guangzhou, China; ^6^Department of Laboratory Medicine, The Fourth People’s Hospital of Foshan, Foshan, China; ^7^Department of Clinical Laboratory, The First People’s Hospital of Foshan, Foshan, China; ^8^Department of Infectious Diseases, Guangzhou Eighth People’s Hospital, Guangzhou Medical University, Guangzhou, China; ^9^Department of Pulmonary and Critical Care Medicine, Shunde Hospital of Guangzhou University of Chinese Medicine, Foshan, China; ^10^The Third Affiliated Hospital of Shandong First Medical University (Affiliated Hospital of Shandong Academy of Medical Sciences), Jinan, China; ^11^Innovative Institute of Chinese Medicine and Pharmacy, Shandong University of Traditional Chinese Medicine, Jinan, China

**Keywords:** COVID-19, T-cell receptor, asymptomatic patient, re-detectable positive case, immune repertoire sequencing

## Abstract

The prevention of the COVID-19 pandemic is highly complicated by the prevalence of asymptomatic and recurrent infection. Many previous immunological studies have focused on symptomatic and convalescent patients, while the immune responses in asymptomatic patients and re-detectable positive cases remain unclear. Here we comprehensively analyzed the peripheral T-cell receptor (TCR) repertoire of 54 COVID-19 patients in different courses, including asymptomatic, symptomatic, convalescent, and re-detectable positive cases. We identified a set of V–J gene combinations characterizing the upward immune responses through asymptomatic and symptomatic courses. Furthermore, some of these V–J combinations could be awakened in the re-detectable positive cases, which may help predict the risk of recurrent infection. Therefore, TCR repertoire examination has the potential to strengthen the clinical surveillance and the immunotherapy development for COVID-19.

## Introduction

As a highly infectious virus, the severe acute respiratory syndrome coronavirus 2 (SARS-CoV-2) caused the pandemic of coronavirus disease 2019 (COVID-19) (1, 2). The clinical manifestations of infected patients ranged from asymptomatic condition to severe symptoms (3). Moreover, among the COVID-19 convalescent patients, some tested positive again after discharge (i.e., re-detectable positive) (4).

The global efforts to end COVID-19 are complicated by the prevalence of asymptomatic and recurrent infection. Many previous immunological studies have focused on symptomatic and convalescent patients (5–12), while the immune responses in asymptomatic patients and re-detectable positive cases remain unclear (13, 14). Unlike symptomatic patients who can be effectively identified by clinical features, asymptomatic carriers may inadvertently transmit virus to close contacts and reshape the dynamics of infection in population (15, 16). Although most re-detectable positive cases have minor symptoms and hardly disease progression upon readmission, their potential infectivity and immunological characterization remain undefined (17, 18). We performed a comprehensive analysis of the transcriptomic profiles of PBMCs from COVID-19 patients in our previous studies (19). However, the pathogenesis is not fully understood at present.

The antiviral adaptive immunity is greatly dependent on the activation of T-cells, which can selectively eliminate virus-infected host cells (20). The specificity toward viral antigens is determined by the structure of the T-cell receptor (TCR) repertoire (21). Based on the advances in sequencing technologies, the biased TCR repertoire in various infectious diseases has been revealed (22–24). Kuri-Cervantes et al. demonstrated the different trajectories of the immunologic state in moderate, severe, and recovered COVID-19 patients (25). Wauters et al. provided deep-immune trajectories of mild to critical COVID-19 by analyzing bronchoalveolar lavage samples (26). Thus, an in-depth study on the TCR characteristics in different courses of COVID-19 is critically needed (27–31).

Here we analyzed the peripheral TCR repertoire of 54 COVID-19 patients in different courses (including asymptomatic, symptomatic, convalescent, and re-detectable positive cases) along with 16 healthy donors. In particular, our results presented the unique immunological features of asymptomatic patients and re-detectable positive cases, which could provide help for clinical management and therapy development.

## Results

### Demographic and Clinical Characteristics

A total of 54 patients with laboratory-confirmed COVID-19 disease were enrolled, including 11 asymptomatic (ASY), 19 symptomatic (SYM), 14 convalescent (CON), and 10 re-detectable positive (RDP) cases. Moreover, 16 healthy donors (HD) were recruited as the control group. As shown in **Table 1**, no significant difference in age or sex was identified between the HD group and any of the patient groups (as measured by the two-tailed Mann–Whitney U-test or Fisher’s exact test). Except for one patient with preexisting chronic pharyngitis, most ASY cases had no obvious clinical symptoms during the whole disease course. All HD subjects and most ASY patients had no underlying comorbidity, while some of the SYM, CON, and RDP patients were diagnosed with hypertension, hyperlipidemia, diabetes mellitus, cardiovascular disease, chronic liver disease, or chronic kidney disease (32). The routine laboratory test results (**Supplementary Table 1**) showed that the lymphocyte levels in the SYM and CON groups were significantly lower than that in the HD group (two-tailed Mann–Whitney U-test p < 0.05). However, such difference was not observed in the ASY or RDP group.

**Table 1:** Baseline characteristics of study subjects.

	Healthy	Asymptomatic	Symptomatic	Convalescent	Re-detectable positive
	(n =16)	(n = 11)	(n = 19)	(n = 14)	(n = 10)
Severity of the first diagnosis (n, %)					
Asymptomatic	0	11 (100%)	0	2 (14.29%)	1(10%)
Mild	0	0	2 (10.53%)	2 (14.29%)	1 (10%)
Moderate	0	0	16 (84.21%)	4 (28.57%)	8 (80%)
Severe	0	0	1 (5.26%)	6 (42.86%)	0
Age (years, median and IQR)	34.50 (27.25–44.25)	33.00 (21.00–44.00)	32.00 (23.00–48.00)	46.50 (23.50–65.75)	30.50 (24.25–59.75)
Male (n, %)	9 (56.25%)	4 (36.36%)	12 (63.16%)	10 (71.43%)	4 (40%)
Female (n, %)	7 (43.75%)	7 (63.64%)	7 (36.84%)	4 (28.57%)	6 (60%)
Comorbidities (n, %)					
Hypertension	0	0	2 (10.53%)	1 (7.14%)	2 (20%)
Hyperlipidemia	0	1 (9.09%)	2 (10.53%)	0	0
Diabetes	0	0	1 (5.26%)	1 (7.14%)	1 (10%)
Cardiovascular disease	0	0	0	1 (7.14%)	0
Chronic liver disease	0	0	2 (10.53%)	1 (7.14%)	1 (10%)
Chronic kidney disease	0	0	0	1 (7.14%)	0
Signs and symptoms (n, %)					
Cough	0	0	13 (68.42%)	4 (28.57%)	1 (10%)
Expectoration	0	0	11 (57.89%)	4 (28.57%)	1 (10%)
Rhinorrhoea	0	0	2 (10.53%)	0	0
Headache	0	0	6 (31.58%)	0	0
Fatigue	0	0	7 (36.84%)	0	0
Sore throat	0	0	3 (15.79%)	0	1 (10%)
Diarrhea	0	0	0	1 (7.14%)	0
Chest tightness	0	0	3 (15.79%)	2 (14.29%)	0
Myalgia	0	0	3 (15.79%)	1 (7.14%)	0
Chills	0	0	4 (21.05%)	1 (7.14%)	1 (10%)
Shortness of breath	0	0	1 (5.26%)	1 (7.14%)	0
Nausea and vomiting	0	0	4 (21.05%)	0	0
Dry throat	0	1 (9.09%)	3 (15.79%)	0	1 (10%)
Gastrointestinal reaction	0	0	1 (5.26%)	0	1 (10%)
Palpitation	0	0	0	0	1 (10%)
Abdominal distension, nausea and retching	0	0	2 (10.53%)	0	0
Poor stomach intake	0	0	1 (5.26%)	0	1 (10%)
Erythra	0	0	1 (5.26%)	0	0
Rhinobyon	0	0	0	0	0

**Figure 1:**
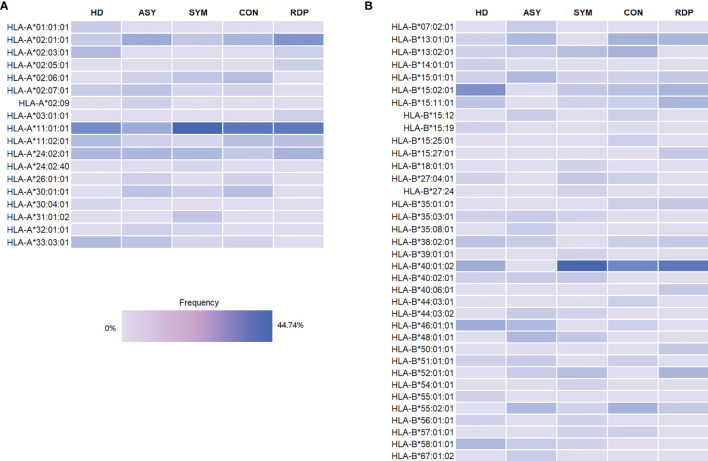
Frequency of HLA-A **(A)** and HLA-A **(B)** alleles in different groups.

Since HLA molecules play a crucial role in shaping the TCR repertoire (33), we also sought to determine whether the frequency of a certain HLA allele was imbalanced across groups. By performing Fisher’s exact test following a previously published procedure (22), we confirmed that none of the HLA alleles showed significant difference (**Figure 1** and **Supplementary Table 2**), which consolidated our following findings in the TCR repertoire.

### Patients in Different Courses Have Heterogeneous TCR Characteristics

**Figure 2:**
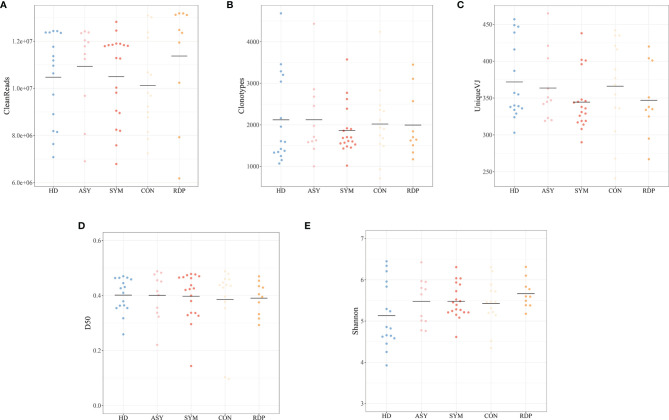
Analysis of data quality and overall clonal diversity among different groups. **(A)** The number of clean reads. **(B)** The number of clonotypes. **(C)** The number of unique VJ pairs. **(D)** Distribution of D50 index. **(E)** Distribution of Shannon entropy.

**Figure 3:**
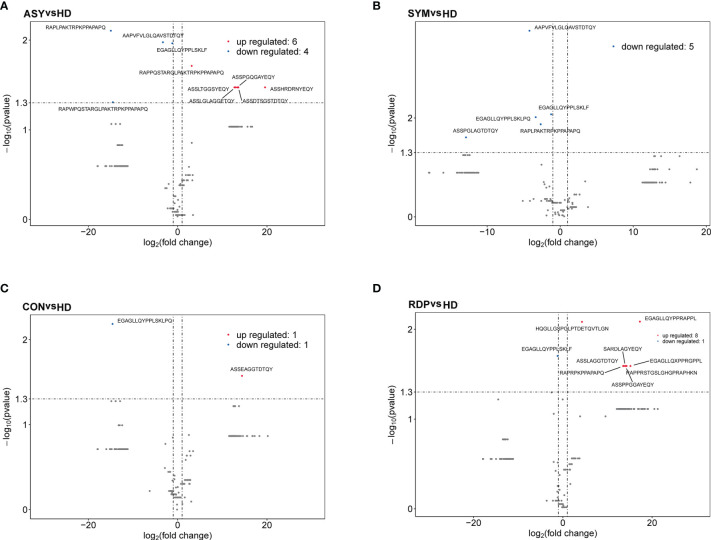
Volcano plots showing differential expression (i.e., |log2(FC)| > 1, p < 0.05) of amino acid clonotypes between patients and healthy donors. **(A)** ASY vs. HD. **(B)** SYM vs. HD. **(C)** CON vs. HD. **(D)** RDP vs. HD.

On average, 10.6 million clean reads were obtained from each sample in sequencing (ranging from 6.2 to 13.2 million, see *Methods*). No significant difference was found between groups in number of reads, clonotypes, or V–J genes (**Figures 2A–C**). No significant difference in clonal diversity (as measured by D50 index and Shannon entropy) was detected between any two groups (**Figures 2D, E****)**, suggesting that COVID-19 might not necessarily induce a widespread change in TCR repertoire diversity. Also, we analyzed the differential expression of amino acid clonotypes in patients and healthy donors (**Figure 3**). The clonotype AAPVFVLGLQAVSTDTQY was significantly decreased in both SYM and ASY groups. Moreover, the clonotype EGAGLLQYPPLSKLF showed a significantly lower expression in both CON and RDP groups.

**Figure 4:**
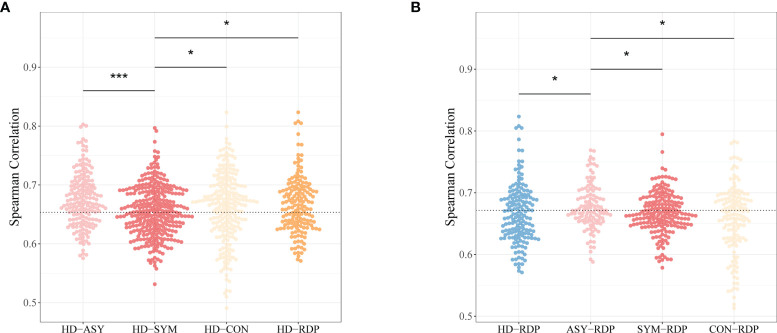
Spearman’s correlation coefficients of V–J combination profiles between different groups. **(A)** The HD-ASY similarity is significantly higher than that of HD-SYM. **(B)** ASY showed the highest similarity to RDP among all groups. Statistical significance of the difference between groups is denoted as * for P < 0.05, *** for P < 0.001.

In the context of V–J gene usage, the similarity between subjects was measured by the Spearman’s correlation (i.e., stronger positive correlation indicates higher similarity) following a previously published procedure (34). Taking HD as a reference, the highest discrepancy was found between SYM and HD. On the other hand, ASY showed significantly higher similarity to HD (**Figure 4A**), suggesting that asymptomatic patients may be subject to less changes in cellular immunology.

Furthermore, taking RDP as a reference, ASY showed the highest similarity as compared to other patient groups (**Figure 4B**). This finding is consistent with the fact that most RDP patients, just as ASY ones, exhibited no obvious clinical symptoms.

### Dynamics of Gene Usage in Different Courses of COVID-19

**Figure 5:**
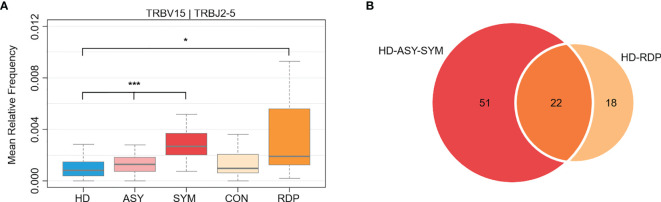
Differential V–J gene usage between different groups. **(A)** An exemplary V–J combination showing a monotonic increase through HD, ASY, and SYM courses, as well as a significant elevation in RDP group. **(B)** Among the monotonic V–J combinations, many were also differentially expressed in RDP cases. Statistical significance of the difference between groups is denoted as * for P < 0.05, *** for P < 0.001.

We further analyzed the dynamics of specific V–J gene expression to identify potential key factors associated with different courses of COVID-19. Using the expression level in HD as a baseline, we identified 73 V–J pairings that exhibited not only alteration in ASY, but even more drastic changes in SYM (Cuzick’s test for monotonic trend p < 0.05, **Figure 5A** and **Supplementary Table 3**) (35). Of note, a significant portion (30.1%, hypergeometric test p = 1.08 × 10^-13^) of the monotonic V–J pairings also showed differential usage in RDP course (RDP vs. HD, Mann–Whitney U test p < 0.05, **Figure 5B** and **Supplementary Table 4**). The dynamics of these feature V–J pairings indicated that a certain immunological memory for SARS-CoV-2 could be awakened upon recurrent infection.

## Discussion

Asymptomatic and recurrent infection further increased the difficulty of COVID-19 prevention. Here we presented an immunological landscape of COVID-19 patients in different courses by performing TCR repertoire sequencing. In particular, while a series of prior studies focused on the immunological profiles of moderate, severe, or recovered patients, our results provided insights into the characteristics of ASY and RDP patients.

Despite numerous recent studies on COVID-19 patients, our analysis adds understanding of the immune responses to SARS-CoV-2 in several aspects. First of all, it has been repeatedly reported that symptomatic patients exhibit more severe clinical manifestations than asymptomatic ones (13, 36–38). In agreement with such observation, we found that the usage of certain V and J gene segments exhibited a monotonic trend through HD, ASY, and SYM groups, indicating potential upward immune responses from asymptomatic to symptomatic course. The V–J combinations showing monotonic changes can help narrow down the search scope of TCR specific to SARS-CoV-2 antigens, which may facilitate the development of highly targeted immunotherapies, such as engineered T-cells for infusion (39, 40).

Next, the risk factors of recurrent infection are not yet well defined (14), which urges development of useful biomarkers to enhance clinical surveillance. We found that a proportion of V–J combinations evoked by asymptomatic and symptomatic infection also changed in re-detectable positive cases. Such observation indicated the possibility of examining immune repertoire as a stringent criterion of complete recovery and an early predictor of high-risk individuals prone to recurrent infection.

Several limitations of our study should be taken into consideration. Firstly, four COVID-19 courses were characterized by blood samples collected from different individuals, rather than sequential samples from the same set of patients. Therefore, additional longitudinal studies are needed to calibrate the immunological trajectories of COVID-19. Secondly, we enrolled a limited number of patients, which makes it difficult to account for factors (e.g., diverse therapies and certain comorbidities) that may have divergent immunomodulatory effects. Subsequent efforts will optimally require larger patient cohorts to control for variations in treatment protocol and prevalence of comorbidities.

Altogether, this study provided novel insights into the TCR profiles of COVID-19 patients in different courses. A better understanding of the adaptive immunity in COVID-19 could strengthen clinical surveillance and immunotherapy development. Further large cohort studies on immune repertoire (41, 42) would be required to extend our findings.

## Methods

### Patients

This study was approved by the ethics committees of the above four participant hospitals. Written informed consent was obtained from all participants enrolled in this study. Between March and May in 2020, a total of 54 patients with laboratory-confirmed COVID-19 disease were enrolled at the Guangzhou Eighth People’s Hospital, the Shunde Hospital of Guangzhou University of Chinese Medicine, the Fourth People’s Hospital of Foshan, and the First People’s Hospital of Foshan, China. The COVID-19 patients were classified into four groups: asymptomatic group, symptomatic group, convalescent group, and re-detectable positive group.

According to the Protocol for Prevention and Control of COVID-19 (Edition 6) of National Health Commission of China (43), the asymptomatic group were those who had a positive COVID-19 RT-PCR test or specific serum IgM antibodies, diagnosed as COVID-19, but without self-perception or clinically recognizable symptoms during the whole disease course. The symptomatic group had evident clinical symptoms and were diagnosed as COVID-19 according to the Diagnosis and Treatment Protocol for Novel Coronavirus Pneumonia (Version 7) of National Health Commission of China (44). In the convalescent group, patients were those with body temperature that returned to normal for more than 3 days, respiratory symptoms significantly improved, inflammation on pulmonary obviously absorbed, and nucleic acid tests of respiratory tract samples proved negative for two consecutive times (sampling interval being at least 24 h). The re-detectable positive group was defined as patients with re-positive results of SARS-CoV-2 nucleic acid during the follow-up period after discharge from the hospital. The time between the re-positive results of the PCR test and the discharge time from the hospital ranged from 2 weeks to 1 month. Besides, 16 health donors were all enrolled at Shunde Hospital of Guangzhou University of Chinese Medicine, China, who tested negative for SARS-CoV-2 and exhibited no respiratory symptoms. Patient demographics and clinical manifestations were retrospectively reviewed. All patients had routine laboratory investigations, including complete blood count, liver function tests, blood gases analysis, and coagulation tests.

According to the Diagnosis and Treatment Protocol for Novel Coronavirus Pneumonia (Version 7) of China, the severity of disease was as follows: (1) mild cases displayed clinical symptoms with no imaging manifestations of pneumonia; (2) moderate cases were characterized by fever and respiratory symptoms with radiological manifestations of pneumonia; (3) severe cases must meet one of the following criteria: respiratory distress (≥30 breaths/min), oxygen saturation ≤ 93% at rest, or arterial partial pressure of oxygen (PaO2)/fraction of inspired oxygen (FiO2) ≤ 300 mmHg (l mmHg = 0.133 kPa).

### RNA Extraction and cDNA Synthesis

Peripheral venous blood was collected and placed into the vacutainer tube. The time points of sample collection are shown in **Supplementary Table 5**. Peripheral blood mononuclear cells (PBMCs) are isolated from 2~4 ml human peripheral blood by Ficoll-Paque density gradient. Total RNA was isolated from PBMCs using TRIzol reagent (Invitrogen, Carlsbad, California, USA) according to the manufacturer’s instruction (miRNeasy Mini Kit, Qiagen, Germany). The RNA quality inspection adopts Agilent 2100, and the quality control standard is RIN > 7.0, 28S/18S ≥ 1.0.

TCR cDNA libraries for high-throughput sequencing were prepared by 5′ rapid amplification of cDNA ends (RACE) using the SMARTScribe™ Reverse Transcriptase (Clontech, Mountain View, California, USA) as previously described (45, 46). Briefly, 0.6 µg of total RNA was mixed with the primer BC1R (**Supplementary Table 6**), which is specific for human TCRβ cDNA synthesis. To denature RNA and anneal the priming oligonucleotides, RNA was incubated at 70°C for 2 min and then at 42°C for 3 min. Switch_oligo and SMARTScribe reverse transcriptases were added for 25 µl template switching and the cDNA synthesis reaction, which was performed at 42°C for 60 min. 5U uracil DNA glycosylase (UDG) was added for digestion at 37°C for 40 min, and the product was purified with MinElute PCR Purification Kit (Qiagen, Germany).

### TCR Library Preparation

Two-round PCR was performed for TCR library preparation. For the first round of PCR amplification, 45 µl of cDNA from the synthesis reaction was mixed with primers and Q5^®^ High-Fidelity 2X Master Mix (NEB, USA). The PCR program began with an initial denaturation at 95°C for 1.5 min, followed by 18 cycles of denaturation at 95°C for 10 s, annealing of primer to DNA at 60°C for 20 s, and extension at 72°C for 40 s, and ended with an extension at 72°C for 4 min. For the second round of PCR amplification, the product from the first round of PCR was purified by QIAquick PCR Purification Kit (Qiagen, Germany), and 10 µl of the purified product was used in each 25-µl PCR reaction. The reaction was performed for 14 cycles using the first-round PCR temperature regimen. PCR products were purified using the QIAquick PCR Purification Kit (Qiagen, Germany). Illumina adaptors were ligated using NEBNext^®^ Ultra™ II DNA Library Prep Kit for Illumina^®^ (New England Biolabs, USA) according to the manufacturer’s protocol and sequencing in the Illumina NovaSeq 6000 platform with the PE150 mode. The parameters of sequencing quality were shown in (**Supplementary Table 7**).

### Data Processing and Analysis

The original data obtained from high-throughput sequencing were converted to raw sequence reads by base calling, and the results were stored in FASTQ format. Low-quality reads and reads without primers were discarded. PCR and sequencing errors were corrected by unique molecular identifiers (UMIs). The reads with same UMI were one clone. Only duplicate reads with different UMIs will be kept in downstream processing. A clonotype was defined by the CDR3 amino acid sequence for further analysis (the same UMI reads must be more than two). TCRβ V, D, and J genes and clonotype were defined according to IMGT59 and IgBLAST; TCRβ VDJ combination was defined by MiXCR. We performed mRNA-Seq profiling on 70 samples. Reads were mapped to the IPD-IMGT/HLA database and HLA-HD determining HLA alleles using mRNA-seq results (47).

### Statistical Analysis

Categorical variables were described as count (%), and a two-group comparison was performed using Fisher’s exact test. Continuous variables were expressed as median and interquartile range (IQR) values and compared by the Mann–Whitney U test between groups. The Spearman correlation coefficient was calculated to assess the association between two vectors of quantitative variables. The significance of monotonic trend across multiple groups was assessed by Cuzick’s test (35). The significance of overlap between two gene sets was assessed by hypergeometric test (the “dhyper” function of R software). All statistical analyses were performed using R software (version 4.0.2). A two-sided p-value lower than 0.05 was considered as statistically significant.

## Data Availability Statement

The raw data supporting the conclusions of this article will be made available by the authors, without undue reservation.

## Ethics Statement

This study was approved by the ethics committees of the above four participant hospitals. Written informed consent was obtained from all participants enrolled in this study.

## Author Contributions

JX, KW, and DW conceived and supervised the project. YS, DL, JL, and WH provided COVID-19 blood samples and clinical information. YuL, JH, DL, YW, and JZ performed the experiments and conducted the sample preparation. KW, YiL. JH, YW, YuL, and WF performed the data analysis. HH, WW, and LL coordinated the projects. YiL, JH, and KW wrote the manuscript. All authors contributed to the article and approved the submitted version.

## Funding

This study was funded by the Science and Technology Innovation Project of Foshan Municipality (2020001000431), National Key Research and Development Project (2020YFA0708001), and Guangdong Provincial Science and Technology Project (2020A1414010363).

## Conflict of Interest

Authors YW, WF and LL were employed by company Guangzhou Huayin Medical Laboratory Center Ltd.

The remaining authors declare that the research was conducted in the absence of any commercial or financial relationships that could be construed as a potential conflict of interest.

## Publisher’s Note

All claims expressed in this article are solely those of the authors and do not necessarily represent those of their affiliated organizations, or those of the publisher, the editors and the reviewers. Any product that may be evaluated in this article, or claim that may be made by its manufacturer, is not guaranteed or endorsed by the publisher.
